# Impact of work hour restrictions on the operative experience of general surgical residents: A systematic review

**DOI:** 10.1016/j.sipas.2023.100222

**Published:** 2023-10-17

**Authors:** Hamza Ashraf, Deepika Gunda, F. Hamish Morgan, Gizem Ashraf, Alexander R. Cortez, Vijayaragavan Muralidharan, Sean Stevens

**Affiliations:** a Department of Surgery, Austin Hospital, Victoria, Australia; b Department of Surgery, Albury-Wodonga Health, New South Wales, Australia; c Centre for Eye Research Australia, Royal Victorian Eye and Ear Hospital, Victoria, Australia; d Department of Surgery, University of California, San Francisco, California, United States; e Surgical Education Research Group, Department of Surgery, Austin Precinct, University of Melbourne, Victoria, Australia

**Keywords:** Work hour restrictions, Resident duty hours, Postgraduate surgical training, Operative experience, Surgical training, General surgery

## Abstract

**Introduction:**

Work hour restrictions (WHR) have been implemented globally to improve resident safety and welfare, but many in the surgical community have raised concerns regarding the impact on resident education and operative experience. This systematic review aims to investigate the impact of WHR on general surgical resident operative caseload.

**Materials and methods:**

Medline and Embase databases were systematically searched according to PRISMA guidelines to identify articles published between 2003 and 2022 assessing the impact of WHR on the operative experience of general surgical residents. Articles were only included if they reported on quantitative measures of operative experience and examined operative caseload after the introduction of WHR.

**Results:**

Of 1,266 studies identified, 26 studies were included, with the majority from US literature. Total major cases underwent a 0.6 % decrease after the introduction of WHR, with surgeon chief cases undergoing a 3.3 % decrease and surgeon junior cases undergoing a 3.7 % increase. Operative trauma underwent the greatest decrease at 18.4 %. Skin & soft tissue cases underwent the greatest increase at 67.6 %.

**Discussion:**

While WHR were often associated with reduced operative caseload in the early years following implementation, the majority of studies found a significant reduction was avoided in the long-term as training programs likely adapted to the new environment. These findings are of particular significance to countries considering the introduction of WHR for surgical residents and may guide future policy and decision-making.

**Conclusions:**

This review demonstrated no significant change in total major cases and an increased caseload for most operative subcategories after the introduction of WHR for general surgical residents. These findings are in keeping with a previous review published in 2011.

## Highlights

• Work hour restrictions (WHR) may impact surgical resident operative experience.
• This study showed there is no significant change in total major cases post-WHR.
• Most studies showed a significant reduction in caseload was avoided long-term.
• These findings may guide policy making in countries considering WHR.

## 1 Introduction

The death of 18-year-old Libby Zion in 1984 and the ensuing highly publicised investigation and trial marked a pivotal moment in the inception of resident work hour restrictions (WHR). [1], [2], [3], [4] Drawing an association between long work hours and poor patient outcomes, in 1989 New York state restricted residents to working no more than 80 h per week. [3] The Institute of Medicine's 1999 ‘To Err Is Human’ report, which attributed 44,000 of 98,000 deaths in United States hospitals to human error, drew further attention to the issue. [5] Thereafter, national mandates followed. The 80 hour per week restriction became nationalised in the US in 2003 following policy reform by the Accreditation Council for Graduate Medical Education (ACGME). These restrictions were extended in 2011 to limit the work hours of first year residents to 16 h per day. [1,2] In 2009, the European Union introduced the European Working Time Directive (EWTD), limiting residents to 48 h per week to improve “workers' safety, hygiene and health at work”. [1,2,[6], [7], [8]] In 2012, Quebec instituted restrictions of 16 hour shifts for all in-house residents. [1,2] At the same time, the Australian state of Queensland introduced similar guidelines for fatigue management in 2011, with recommendations that all shifts should be limited to a maximum of 16 h per day. [9,10]

Since their implementation, WHR have faced criticism, particularly in surgery where long hours at work have traditionally been regarded as necessary for obtaining clinical competence in operative technique. [7,11,12] Views on WHR range from extoling resident safety and well-being through to condemning the limitations on education and surgical training progression. [1,3,4,7,8,[11], [12], [13], [14], [15]] Moreover, whether WHR have achieved the desired outcome of improving patient outcomes is also debated. Prior work evaluating the impact of WHR in the US has found preserved educational outcomes and improvement in resident well-being. [1,2] However, reliable data to inform such discussion is difficult to obtain, given the impracticality of controlled trials. [16,17] Potential confounders in observational studies, such as the impact of hospital quality improvement programs, also make interpretation of this data difficult. [4]

Currently, not all countries have regulations around surgical resident working hours, or indeed around working hours at all. [18] For example, while New Zealand has restricted surgical residents to working a maximum of 72 h per week since 1985, Australia has no such restrictions, despite both countries conducting a joint surgical training program. [19,20] There is continuing concern in the surgical community that reducing surgical resident weekly hours may significantly prolong training time to achieve the required competency or result in accepting a lower standard of competency. [7,8,11,12,14] Moreover, training boards around the world have transitioned, or are considering transitioning, from time-based to competency-based or hybrid training programs, emphasising the importance of better understanding the impact of work hours on operative experience and learning. [21]

It is important to evaluate the impact of WHR in surgery to inform educators and policy makers to help guide future policy making. Assessing resident competence, confidence and autonomy is complex and requires evaluation of technical and non-technical competencies. Operative caseload can be measured objectively and has traditionally served as the mainstay of a graduate's operative experience. Despite the emergence of new assessment tools and their increasing use, [22] operative caseload remains a useful measure of exposure and training opportunities. This systematic review aims to investigate the impact of WHR on general surgical resident operative caseload.

## 2 Material and methods

### 2.1 Search strategy

A systematic review was performed in accordance with Preferred Reporting Items for Systematic Reviews and Meta-Analyses (PRISMA) guidelines. Two online databases, Embase Classic and Ovid MEDLINE(R) Epub Ahead of Print, In-Process & Other Non-Indexed Citations, were searched to identify articles related to the impact of WHR on operative experience of general surgical residents.

Search terms were developed in conjunction with experts in surgical education. The final search strategy explored three broad concepts: general surgery, WHR, and resident operative caseload (see Appendix 1). Search terms were adapted to relevant subject headings of each database (e.g. MeSH for Medline and EMTREE for Embase). Search terms within the same concept were combined with the Boolean operator 'OR', then the three concepts were combined with the Boolean operator 'AND'.

### 2.2 Eligibility criteria

Criteria for inclusion comprised studies which focused on work hours or WHR with respect to operative experience of general surgical residents, with a study design including observational studies, prospective and retrospective reviews, cohort studies and randomised controlled trials. Studies were only included from 2003 to 2022, thereby limiting studies to those investigating the effects of major WHR implementations around the world.

Pre-defined criteria were used to exclude articles beyond the scope of this review. Studies were excluded if they did not include all of the following three criteria – reporting on general surgical training data, reporting measures of operative experience (e.g. number of operations performed), and investigating the impact of work hour restrictions. Studies were excluded if operative caseload data was not available for extraction. Studies involving operative experience of medical students or junior residents (i.e. not affiliated with a dedicated general surgery program) or where residents were involved in the operation in an assisting role only were also excluded. Study designs not eligible included case studies, surveys, questionnaires, interviews, focus groups, letters, editorials and systematic reviews and meta-analyses.

### 2.3 Article selection

**Fig. 1:**
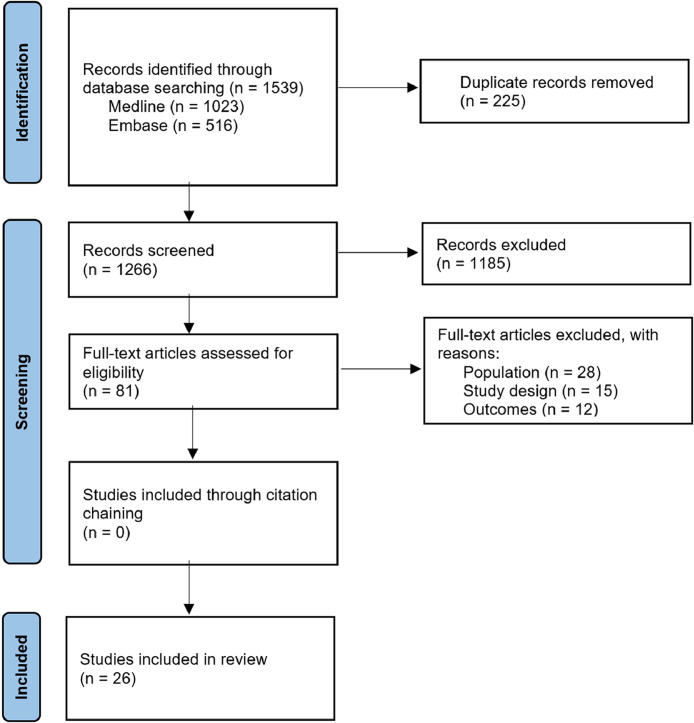
PRISMA flow diagram for article selection.

All references were managed by EndNote™ X9. The articles identified by the search strategy were collated and duplicates excluded. Unique articles were screened for eligibility initially by title and abstract by two members of the research team (H.A. and G.A.), with conflicts resolved through consensus. Full-text screening of the articles was independently performed by two members of the research team (H.A. and D.G.). Any unresolved differences in study inclusion were referred to a senior researcher (S.S.) for determination. If an article was not available online or through a library database, authors were contacted to request access to the article. Non-English language articles were translated using Google Translate. The article selection process is summarised using the PRISMA flow diagram (Fig. 1).

### 2.4 Data extraction

Microsoft Excel was used to develop the data extraction tool. Data extraction was performed by two members of the research team (H.A. and D.G.). Data extracted included study year, study design, country, cohort demographics including work hours, and quantitative data for operative experience including total major cases, surgeon chief and surgeon junior cases, and operative subcategory caseload. Authors were contacted to request missing or incomplete data. Data was only included if absolute values of operative cases were available.

### 2.5 Data analysis

Extracted data was modified and synthesised to a tabular format for interpretation. Data analysis was performed by two members of the research team (H.A. and D.G.). The Joanna Briggs Institute (JBI) Critical Appraisal Checklist was used to assess quality and risk of bias by two researchers (H.A. and D.G.). [23] Unresolved conflicts were referred to a third member of the research team (S.S.) for resolution. Articles with a ‘yes’ percentage of >70 % were graded as high quality, those with a ‘yes’ percentage of 50–69 % were graded as moderate quality, and those with a ‘yes’ percentage of <50 % were graded as low quality. [23]

Weighted percentage change in caseload was calculated for each operative subcategory analysed by a study, using the formula below. The study weighting was calculated by dividing the study's sample size by the total sample size.

$$\frac{Study\;sample\;size}{Total\;sample\;size}=Study\;weighting$$

The study weighting was multiplied by the percentage change in caseload reported by that study to calculate the weighted percentage change in caseload for that study.

Studyweighting×Studypercentagechangeincaseload=Weightedpercentagechangeincaseloadforstudy

The weighted percentage change in caseload for all studies were totalled to determine the mean weighted percentage change for the operative subcategory.

Weightedpercentagechangeincaseloadforstudy1+weightedpercentagechangeincaseloadforstudy2+…+weightedpercentagechangeincaseloadforstudyx=meanweightedpercentagechangeincaseload

**Fig. 2:**
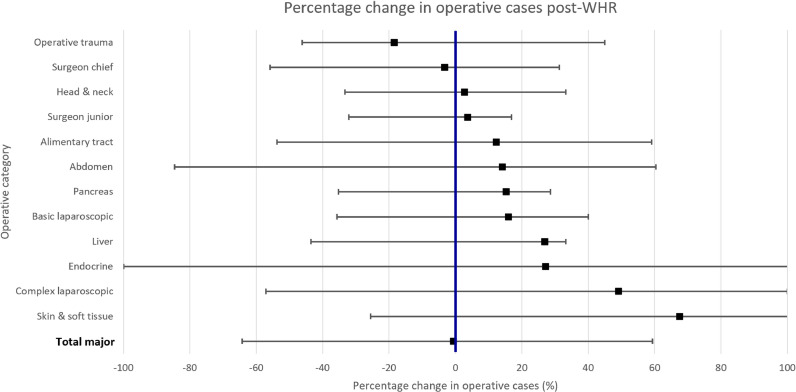
Change in resident operative caseload after introduction of work hour restrictions (WHR) Results are reported as mean weighted percentage change in operative caseload after WHR. The lower and upper limits of the error bars represent the single study demonstrating the greatest decrease and increase, respectively.

Studies which did not report a sample size were not included in these calculations, and therefore were not included in the creation of Fig. 2.

## 3 Results

The literature search yielded 1266 unique abstracts, with 81 full-text studies assessed for eligibility (Fig. 1). From this, 55 studies were excluded due to ineligible study design, population group (e.g. did not include general surgical resident data) or outcome evaluation (e.g. did not include operative caseload data). Of the 26 included studies, 23 analysed US data and three analysed European data. Further study characteristics are outlined in Table 1. Quality assessment was performed using the JBI Critical Appraisal Tool and all 26 studies were rated as high quality.

**Table 1:** Characteristics of included studies Resident numbers were not available (NA) in seven studies.

First author	Publication year	Location	Data period	Sample size (residents)	Study type
Blencowe [41]	2011	Europe	1996–2009	NA	Single institution
Breen [42]	2013	Europe	2008–2009	12	Single institution
Hopmans [48]	2015	Europe	2005–2012	64	Multi-institution
Feanny [28]	2005	USA	2001–2004	13	Single institution
Ferguson [29]	2005	USA	2002–2003	NA	Single institution
De Virgilio [30]	2006	USA	1998–2005	NA	Single institution
Tran [31]	2006	USA	2000–2004	NA	Single institution
Carlin [32]	2007	USA	2001–2005	120	Single institution
Damadi [33]	2007	USA	2000–2005	17	Single institution
Durkin [34]	2008	USA	1997–2005	NA	Single institution
Christmas [36]	2009	USA	2002–2008	28	Single institution
Bruce [37]	2010	USA	2001–2009	29	Single institution
Watson [40]	2010	USA	1988–2008	40	Single institution
Scally [46]	2014	USA	2005–2013	45	Single institution
Condren [47]	2015	USA	2008–2013	109	Single institution
Quillin [49]	2016	USA	1992–2015	135	Single institution
Cortez [52]	2018	USA	1999–2017	106	Single institution
Schwartz [45]	2013	USA	2007–2012	249	Multi-institution
Kairys [35]	2008	USA	1992–2006	NA	National
Fairfax [38]	2010	USA	1999–2008	2013	National
Simien [39]	2010	USA	2002–2008	6049	National
Drake [43]	2013	USA	1989–2012	23,334	National
Patel [44]	2013	USA	1990–2010	NA	National
Drake [50]	2017	USA	1989–2013	24,432	National
Strumwasser [51]	2017	USA	1999–2015	2053	National
Cortez [53]	2018	USA	1990–2016	27,851	National

Seven out of 26 included studies did not report sample size. Studies were still included even if they did not report a sample size (number of residents), as long as data for mean operations per resident was available. This data is independent of sample size and is reported in Table 2 and Table 3. However, studies which did not report sample size were not included in the creation of Fig. 2, as it was not possible to adjust the effect size of the study based on sample size.

**Table 2:** Resident operative caseload before and after introduction of work hour restrictions (WHR) [colour] Results are reported as mean number of major operative cases per resident, with the first value representing pre-WHR cases and the second value representing post-WHR cases. Studies published in the US report pre-WHR and post-WHR values relative to either WHR introduced in 2003 or 2011, whereas European studies report values relative to WHR introduced in 2009. ↑ (green) represents a statistically significant increase in operative cases, ↓ (red) represents a statistically significant decrease and ↔ (yellow) represents no statistically significant difference. (E) denotes European studies. * denotes studies which reported operative data for a 1-year period only. † denotes studies which reported operative data for a 2-year period only.^#^*denotes studies which utilised measures other than mean.*

**Table 3:** Resident operative caseload before and after introduction of work hour restrictions (WHR) for operative subcategories [colour] Results are reported as mean number of major operative cases per resident, with the first value representing pre-WHR cases and the second value representing post-WHR cases. Studies published in the US report pre-WHR and post-WHR values relative to either WHR introduced in 2003 or 2011, whereas European studies report values relative to WHR introduced in 2009. ↑ (green) represents a statistically significant increase in operative cases, ↓ (red) represents a statistically significant decrease, ↔ (yellow) represents no statistically significant difference and X (grey) represents where statistical significance was not analysed. (E) denotes European studies. ^#^ denotes studies which utilised measures other than mean.

Of the 26 included studies, 24 studies evaluated operative experience for total major cases, surgeon chief cases and surgeon junior cases, as listed in Table 2. Eighteen studies analysed data for total major cases. Of the two European studies analysing total major cases, one study reported a decrease whereas the second study reported no change in operative caseload. Of the 16 US studies, only one study reported an increase in operative volume, whereas eight studies reported a decrease and seven studies reported no change in operative volume.

Fourteen studies analysed data for operative volume as surgeon chief (final year surgical resident). The single European study demonstrated a decrease in operative volume. Of the 13 US studies, only one study demonstrated an increase in operative volume as surgeon chief, with five studies reporting a decrease and seven studies reporting no change.

Seven studies analysed data for operative volume as surgeon junior (all non-surgeon chief resident years). The single European study demonstrated no change. Of the six US studies, one study demonstrated an increase in cases as surgeon junior, with three studies reporting a decrease and two studies reporting no change.

Sixteen out of 26 included studies utilised more granular analyses of operative case data for specific operative subcategories (Table 3). Given the large range of sample sizes in the studies, and to avoid giving equal weighting to smaller and larger studies, the formula described in the methods section was used to calculate the mean percentage change in operative caseload for each operative subcategory (Fig. 2) – this adjusted the effect size of the study based on its sample size.

For example, Simien et al. had a sample size of 6049 residents, resulting in a study weighting of 9.9 %. Simien et al. demonstrated a 2.8 % decrease in total major cases after WHR. Therefore, the weighted percentage change in caseload can be calculated:

Studyweightingof9.9%%x2.8%%reporteddecreaseintotalmajorcases=weightedpercentagechangeincaseloadof0.27%%decrease

This designates the contribution of Simien et al. to the overall result. Similar calculations were performed for each study that reported on total major cases, and these were totalled to calculate a mean 0.6 % decrease in total major cases after WHR.

Although the mean percentage change is a useful summary statistic, as Fig. 2 demonstrates there was large variability between studies, ranging from a 63 % decrease in total major cases to a 60 % increase.

## 4 Discussion

WHR have influenced many aspects of resident training, with a unique effect on surgical residents being the impact on resident operative experience. This systematic review examines the impact of WHR on general surgery resident operative experience, and includes ten studies published since a previous systematic review in 2011 examining the impact of the 2003 WHR on all surgical residents. [2] In summary, our review demonstrated no change in total major cases and an increased caseload in most operative subcategories.

A prior review by Jamal et al. from 2000 to 2008 investigated the outcomes of the 2003 ACGME WHR on several surgical specialties, including general, plastic and orthopaedic surgery, with most studies examining general surgery. [2] In regards to the impact of WHR on operative caseload, 18 studies (62 %) demonstrated neutral or positive effects and 11 studies (38 %) demonstrated a negative effect. This is in keeping with our findings, with 11 studies (61 %) demonstrating a neutral or positive effect and seven studies (39 %) demonstrating a negative effect.

While the proportion of studies demonstrating neutral or positive effects may be similar between the two reviews, a novel and important finding of our review is the reversal in trends following the 2011 WHR compared to the 2003 WHR. While a majority of earlier studies in our review (up to and including 2011) found WHR to be associated with a reduction in total major cases (55 %), recent studies have rarely found this association (14 % of studies post-2011), with many instead demonstrating no change in total major cases (86 %). The reasons for this change in trend are unclear but may relate to improved efficiency as residents adapted to the new work hour restrictions and training institutions that were initially negatively impacted by WHR were able to adapt and better provide residents with similar operative exposure despite reduced hours worked.

Another important finding of this study is the preserved caseload for most operative subcategories. Operative trauma underwent the greatest reduction in caseload. This may reflect the trend towards non-operative management of trauma in modern surgery and that a higher proportion of trauma surgery occurs out of hours with WHR likely to cause residents to miss operative opportunities. [24] Similarly, increases in basic and complex laparoscopic surgery likely reflect the increasing incidence of laparoscopic surgery in the past two decades. [25] The greatest increase in caseload was a 68 % increase in skin & soft tissue cases, perhaps contributing to the overall finding of no change in total major cases. This increase in skin & soft tissue cases may reflect a greater proportion of resident caseload shifting to simpler cases, perhaps in part due to an increase in fellowship positions over time, with residents subsequently performing a narrower range of cases. [26,27]

Several studies have explored the impact of WHR on other non-operative aspects of resident education. In 2011, Jamal et al. found that WHR generally improved resident well-being and preserved educational outcomes but were detrimental to surgical faculties. [2] In 2014, Ahmed et al. conducted a systematic review of 57 studies from 1980 to 2013 to additionally investigate the effect of the newer 2011 ACGME WHR. [1] They found similar benefits in resident well-being after the 2003, but not 2011, resident WHR. Educational outcomes were generally either unchanged or reduced in terms of examination performance or self-perception. However, resident operative caseload was not examined. Finally, the effect of WHR on patient safety was regarded as inconclusive. As WHR have consistently been shown to be associated with improved resident well-being, [1,2] and the effect on resident education and patient safety is inconclusive, the overall finding of no change in operative caseload in this study is tentatively considered a positive outcome.

There is a paucity of literature examining WHR and the impact on surgical residents outside the US and Europe. The majority of studies in this review examine US data, with this review exploring the impact of WHR many years after the implementation of both the 2003 and 2011 US WHR. While the impact of these WHR is anecdotally known to most US surgical educators, the findings of this review are important for countries considering or having recently introduced WHR to learn from the US experience. [20] While Australia employs collective bargaining agreements to regulate working hours, as yet there are no maximum limits on working hours, despite a position paper by the Royal Australasian College of Surgeons recommending a maximum 70 hour working week. [12,20] Although working hours in these countries have changed over time, there have been no studies examining the relationship between working hours and general surgical resident operative experience.

It is important to note that the jurisdictional control of WHR is multi-institutional, involving departments of health in national and state governments, national training boards and local hospital committees, making it difficult to compare and translate WHR policies and their impact between countries. Additionally, the structure of medical training programs differs between continents, and it is important to acknowledge the diverse impact that WHR may have on trainees from different countries. For example, USA training programs are graduate entry programs, with WHR impacting residents during the five years of general surgery resident training, but not during the prior undergraduate and medical school years. On the other hand, European training programs are undergraduate entry programs, with WHR impacting trainees during both foundation training years as well as general surgery resident training years.

Several limitations to this study are recognised. Firstly, it is difficult to compare the effects of WHR between countries and caution must be exercised when comparing data, however is it important to note that the majority of included studies were US studies. While a meta-analysis may provide a more comprehensive summary of the available data, this was not performed due to the heterogeneity of the training programs studied. This study only considers operative caseload as an indicator of operative experience and does not take into consideration other measures of operative experience (e.g. case complexity or surgeon mentorship provided). This was because operative caseload was widely reported whereas other indicators of operative experience were not. This study does not examine trainee wellbeing, satisfaction or patient safety when considering operative experience. We recognise these outcomes as important and worthy of further investigation.

## 5 Conclusion

This systematic review examines the impact of work hour restrictions on general surgery resident operative experience. Most studies, especially since 2011, demonstrate that total major cases and most operative subcategories have not been adversely affected by the introduction of WHR. Although study findings were inconsistent, our review suggests that while WHR were often associated with reduced operative caseload in the early years following implementation, the majority of studies found a significant reduction was avoided in the long-term as training programs likely adapted to the new environment. These findings are of particular significance to countries considering the introduction of WHR for surgical residents and may guide future policy and decision-making. Finally, the impact on other aspects of resident competencies that require experiential learning remains to be sufficiently evaluated.

## Funding

This research did not receive any specific grant from funding agencies in the public, commercial or not-for-profit sectors.

## Declaration of Competing Interest

The authors declare that they have no known competing financial interests or personal relationships that could have appeared to influence the work reported in this paper.

## Appendix A

Search terms within one concept combined with ‘OR’, then the 3 concepts combined with ‘AND’.

• General surgery
  ○ General Surgery
• Medical education
  ○ Graduate medical education
  ○ Fellowships and scholarships
  ○ Internship and residency
  ○ PGY, postgraduate year
  ○ ACGME, accreditation council on graduate medical education
  ○ Professional competence
  ○ Clinical competence
  ○ Curriculum
  ○ Surgical volume
  ○ Surgical training
  ○ Operative experience
• Work hours
  ○ Personnel staffing and scheduling
  ○ Workload
  ○ Time factors
  ○ Duty hour*
  ○ Working time
  ○ Work schedule
  ○ Work schedule tolerance
  ○ Work hour restriction*
  ○ Duty hour restriction*
  ○ Work hour*
  ○ Restriction*

Appendix A**: Search strategy for OVID Medline**
